# Antitumor effects of fecal microbiota transplantation: Implications for microbiome modulation in cancer treatment

**DOI:** 10.3389/fimmu.2022.949490

**Published:** 2022-09-13

**Authors:** Hui Xu, Chenxi Cao, Yuqing Ren, Siyuan Weng, Long Liu, Chunguang Guo, Libo Wang, Xinwei Han, Jianzhuang Ren, Zaoqu Liu

**Affiliations:** ^1^ Department of Interventional Radiology, The First Affiliated Hospital of Zhengzhou University, Zhengzhou, China; ^2^ Interventional Institute of Zhengzhou University, Zhengzhou, China; ^3^ Interventional Treatment and Clinical Research Center of Henan Province, Zhengzhou, China; ^4^ Department of Respiratory and Critical Care Medicine, The First Affiliated Hospital of Zhengzhou University, Zhengzhou, China; ^5^ Department of Hepatobiliary and Pancreatic Surgery, The First Affiliated Hospital of Zhengzhou University, Zhengzhou, China; ^6^ Department of Endovascular Surgery, The First Affiliated Hospital of Zhengzhou University, Zhengzhou, China

**Keywords:** cancer, fecal microbiota transplantation, FMT, gut microbiota, immunotherapy

## Abstract

Fecal microbiome transplantation (FMT) from healthy donors is one of the techniques for restoration of the dysbiotic gut, which is increasingly being used to treat various diseases. Notably, mounting evidence in recent years revealed that FMT has made a breakthrough in the oncology treatment area, especially by improving immunotherapy efficacy to achieve antitumor effects. However, the mechanism of FMT in enhancing antitumor effects of immune checkpoint blockers (ICBs) has not yet been fully elucidated. This review systematically summarizes the role of microbes and their metabolites in the regulation of tumor immunity. We highlight the mechanism of action of FMT in the treatment of refractory tumors as well as in improving the efficacy of immunotherapy. Furthermore, we summarize ongoing clinical trials combining FMT with immunotherapy and further focus on refined protocols for the practice of FMT in cancer treatment, which could guide future directions and priorities of FMT scientific development.

## Introduction

In 2022, there are expected to be 191,830 new cancer cases and 609,360 cancer deaths in the United States (1), which is increasing year by year (2, 3), consequently, more targeted cancer control interventions in cancer are needed to reduce cancer mortality. Usually, only 5-10% of all cancer cases are considered to be attributed to genetic defects (4), the remaining 90-95% have their roots in the environment and lifestyle that include smoking, diet, alcohol, sun exposure, environmental pollution, infection, stress, obesity and lack of exercise (5, 6). In addition, over the past few decades, the link between microorganisms and the development of several cancers has also been generally recognized. Therefore, it is important to understand the role of microorganisms in tumor prevention and treatment. Helicobacter pylori in gastric cancer (7), the human papillomavirus (HPV) in cervical cancer (8), and the hepatitis B and C viruses (HBV and HCV) in hepatocellular carcinoma (9) are the best evidence that the microbiota is not passenger or bystander. The influence of the gut microbiota on the development of certain tumors and the specific mechanisms have recently received much attention. Bacteria are found in many tissues and organs of the body, especially in the digestive tract, and their numbers and species are constantly changing (10). Host genetics, dietary components, drugs, chemicals, aging and stress have been shown to regulate the dynamic balance of the intra-host microbiome (11, 12). Microbial dysbiosis in the gut has been connected to the development of tumors both inside and outside the gastrointestinal system, including colon cancer, esophageal cancer, liver cancer, and breast cancer (13–15). Strategies against the gut microbiota may influence the course of disease in which dysbiosis is observed. Various approaches, including dietary interventions, the use of probiotics and antibiotics, and fecal microbiota transplantation (FMT) have been used to modulate the gut microbiota to prevent or treat different cancer pathological processes (16). As research on intestinal flora continues to progress, FMT as a novel treatment modality has been used for the restoration of gut microbial dysbiosis.

FMT, which originated in Chinese medicine at least 1700 years ago, is the transfer of fecal microbiota from a healthy donor into the patient’s intestine to rebalance the flora (17). It can target and modulate the human gut microbiota, which has been widely studied in the treatment of various diseases (18). Given that FMT maintains microbial diversity without disrupting the natural balance of the microbial gut, it exhibited significant advantages over other treatment modalities. FMT is commonly used for the treatment of Clostridioides Difficile infections (CDIs) (19, 20). More than that, promising results have indicated that FMT also is effective in other digestive diseases, including colonized with multidrug-resistant organisms (MDROs), inflammatory bowel disease (IBD), and irritable bowel syndrome (IBS), and neurological disorders including multiple sclerosis (MS), hepatic encephalopathy (HE), Parkinson’s disease, and diabetic neuropathy (18, 21–25). Meanwhile, more evidence shows that FMT also has an antitumor effect which will be an emerging treatment modality for cancer such as melanoma (26, 27). The regulatory and antitumor effects of FMT on the host intestinal microbiota are achieved by affecting the immune system and inflammatory response, changing microbial metabolites, affecting cell signaling pathways, inhibiting DNA damage, and acting on extraintestinal parts through blood circulation (28). In this review, we will focus on the role and mechanisms of human gut microbiota in tumor development. Likewise, we will discuss the antitumor effect and clinical application of FMT by modulating or reconstructing intestinal microbiota, particularly in oncotherapy, including immunotherapy, chemotherapy, and radiotherapy.

## The gut microbiota as a target for therapy

Humans first acquire a large microbiota from their mothers at birth, and the microbiota is constantly changing as they grow and develop (29, 30). There is a bidirectional feedback loop between microbiota and host health. On the one hand, many studies revealed significant correlations of the gut microbiome with host genetics, environment, changes in lifestyles, and diet (11, 12, 31). Since the genome of the host can affect the make-up and function of the microbial community, human and mouse genetic motifs are being mapped by genome-wide correlation studies (32). For example, a microbial trial in the Netherlands involving 7,738 individuals examined the association of 207 of these taxa and 205 genome-wide entries representing microbial composition and function and found two signals associated with microbial taxa in the vicinity of the LCT and ABO genes and replicated in two independent cohorts (11). In addition, a long-term specific diet of the host can make some specific microbiota dominant, and some microorganisms may even become extinct (33). In that study, successive generations of rats fed a low-fiber diet revealed a gradual loss of microbiota diversity, with some taxa being undetectable. On the other hand, the complex and unique make-up of the gut microbiome community influences host metabolism, immunity, and elimination of harmful substances (10, 34). Our gut microbiota not only helps us absorb nutrients from our food, but also contributes to the body’s immune system (35). A recent study through mouse models and brain imaging technology has proved that intestinal microorganisms can affect brain function and metabolism (36). As long as the gut microbiota is functioning properly and maintaining a balance (symbiosis), the host physiology is maintained and protective effects are obtained. Moreover, a standard and healthy eubiosis cannot be standardized due to individual differences, and maintaining a balanced microbial composition in one individual is not the best choice for others. Nevertheless, it is almost certain that gut microbiome diversity is associated with human health (37, 38).

In contrast to eubiosis, there is an imbalanced gut microbiome or altered community structure in various disease states, which is called dysbiosis, resulting in the production of large amounts of harmful metabolites. Dysbiosis, implying disturbances in microbial composition and metabolism, is relevant to a wide range of disorders and a therapeutic target (39). For example, lifestyle changes and high-fat diets can cause dysbiosis of intestinal flora, increasing LPS expression levels and decreasing miR-145, leading to metabolic inflammation and metabolic disorders (40, 41). Of note, an imbalance in the gut microbiota can also contribute to the progression of cancer. Dysbiosis implies the production of harmful metabolites by the microbiota as well as causing immune dysfunction in the body, which leads to several diseases such as IBD and IBS. In addition, long-term metabolic disorders and inflammation can lead to the development of tumors (42). For instance, colon cancer may progress from chronic inflammation caused by dysbiosis (15, 43, 44). One very important recent finding indicates that patients with esophageal cancer also had dysbiosis of the gut microbiota (n = 10) (13).

## Microbial mechanisms of tumorigeneses and progression

**Figure 1:**
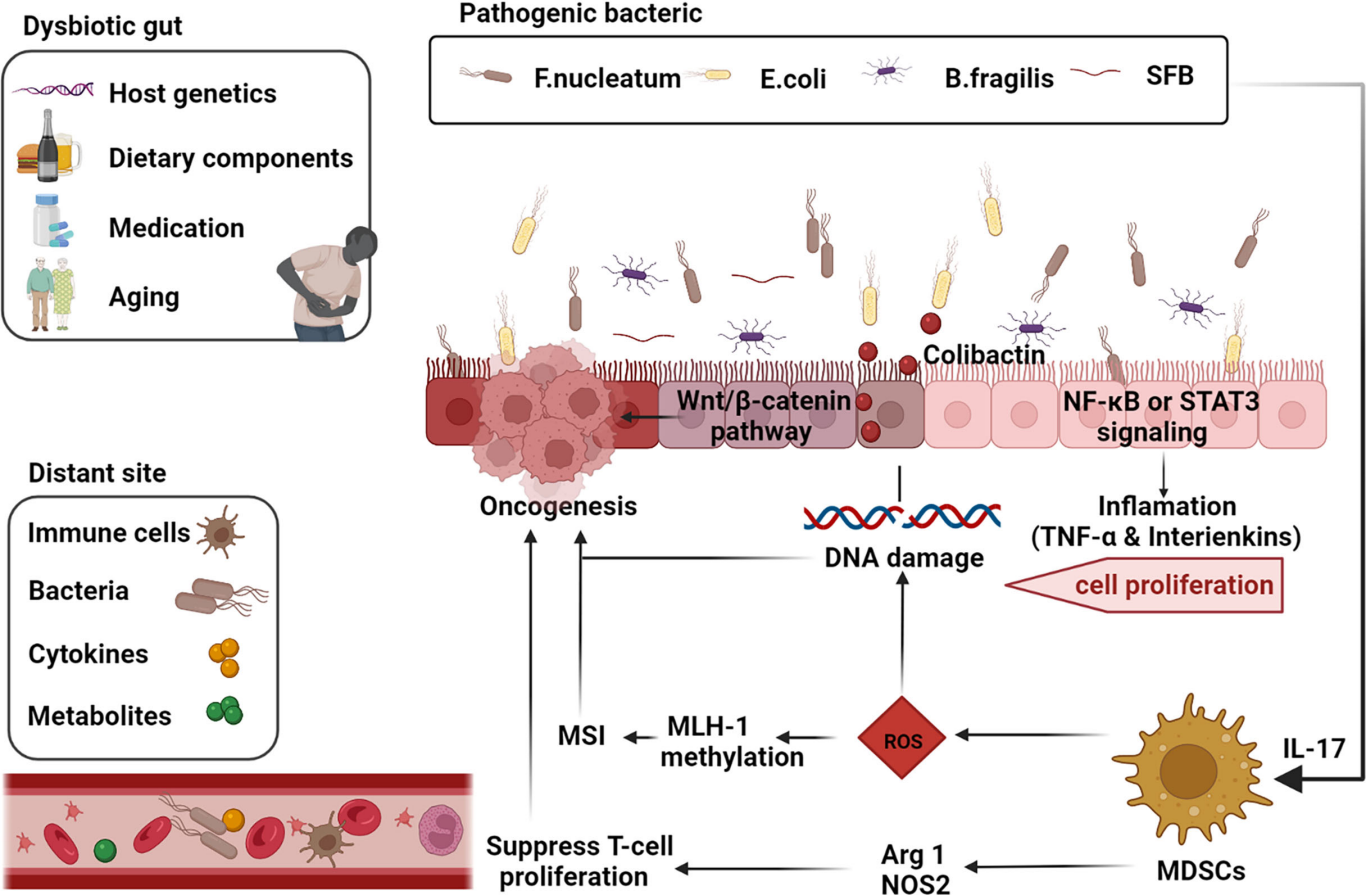
Gut-microbiome-mediated mechanisms of oncogenesis and progression. Factors such as the host genetics, unhealthy dietary components, medication, and aging cause gut dysbiosis. Inflammatory markers such as TNF-α and interleukins such as IL-17 are activated by the corresponding pathogenic microbial strains *via* the NF-κB or STAT3 pathways thereby inducing cell proliferation. Microbial strains such as Segmented filamentous bacteria (SFB) are effective inducers of Th17 cells in the SILP. E. coli induces DNA damage and thus promotes tumorigenesis through the release of virulent substances such as coliphage. Clostridium nucleatum activates the differentiation of myeloid-derived suppressor cells (MDSCs) and further induces reactive oxygen species (ROS), leading to MutL homolog 1 (MLH-1) methylation and microsatellite instability (MSI), leading to tumor progression. Activated MDSCs also inhibit T cell differentiation and promote tumor progression through activation of Arg1 and NOS2-mediated antitumor immunity (adapted from 16). F, nucleatum also stimulates cell proliferation through activation of the Wnt/β-linked protein pathway. Many components of our daily diet are metabolized by bacteria in the digestive tract and produce the corresponding metabolites such as secondary bile acids, which promote carcinogenesis. Furthermore, intestinal microbiota, microbial metabolites, immune cells and cytokines can also produce carcinogenic effects at different sites in the distal part of the body through blood circulation. (The figure was created with Biorender.com).

Intestinal goblet cells maintain the epithelial barrier by secreting mucus (45), which provides a habitat for commensal bacteria and prevents bacterial infiltration and inflammation, thus maintaining barrier integrity (46). The intestinal microbiome is essential in preserving the integrity of the gut barrier (47). Furthermore, the gut microbiota aids digestion, helps with vitamin production and regulates physiological functions (36, 48, 49), including regulation of metabolism, blood production, immune enhancement, and protection against cancer (13–15, 36, 50, 51). Conversely dysbiosis of the microbiota can also promote tumors. In this review, we pay attention to the microbial mechanisms that may be involved in tumorigenesis and progression (**Figure 1**).

### Immune system and inflammation

Chronic inflammation and immune responses are regulated by the gut microbiota and may progress to tumors (52). While laboratory mice reconstituted with natural microbiota improved drug resistance to mutagens/inflammation-induced colorectal tumors resistance (53). IL-10 is primarily known as an immunosuppressive cytokine that promotes cell multiplication and metastasis of tumors (54). A study of conventional IL-10 mice exposed to AOM found that tumor diversity was directly related to the presence of colitis. The colon histology of sterile AOM treated IL-10 mice were normal and there was devoid of tumors. That is, bacterial-induced inflammation promotes the progression from adenoma to invasive cancer. What’s more, this study is the first direct proof that the manipulation of intestinal microbiota changes the progression of colorectal cancer (CRC) (55). Another cytokine, IL-17 is predominately produced by pathogenic proinflammatory Th17 cells and has been implicated in many autoimmune inflammatory disorders (56). Th17 cells are not found in sterile mice and have to be generated by certain subpopulations of specific intestinal microorganisms (57). For example, segmented filamentous bacteria (SFB) is a potent inducer of Th17 cells in the SILP of mice (58, 59). Enterotoxigenic Bacteroides fragilis (ETBF) can not only induce Th17-mediated colitis by producing pathogenic toxins but also activate colon specific signaling transducers and STAT3 to induce tumors (60–62). Furthermore, antibody-mediated IL-17 blockade can inhibit ETBF-induced colitis and tumor development (62).

### DNA damage

DNA damage is a major contributor to cancer and the microbiome causes the induction of carcinogenesis by inducing DNA damage, modulating cell growth and apoptosis, producing epigenetic changes, and regulating host immune responses (63). For example, coliphage and cell lethal swelling toxins cause genomic instability and damage DNA directly (64). Several recently published pieces of research have shown that Colibactin is produced by a cluster of genes in some E. coli called polyketide synthase islands (65, 66). In addition, it produces secondary metabolites, ROS, endogenous sources of which include nicotinamide adenine dinucleotide phosphate oxidase complexes, peroxisomes, and mitochondrial respiration (67, 68). The overproduction of ROS is the key cause of oxidative stress, which affects lipids, proteins, RNA, and DNA, as well as impairing various cellular functions (69, 70). In certain settings, Enterococcus faecalis produces large amounts of extracellular hyperoxides (O2-) in the colonic mucosal lumen (71). H2O2 produced by rapid degradation of O2 can penetrate the cell membrane and damage eukaryotic DNA. Furthermore, Bifidobacterium Fragilis toxin enhances the bacterial polyamine-catabolic pathway and generates reactive oxygen species, which can also damage host DNA and even lead to colon cancer (72).

### Diet and microbial metabolites

Bacteria in the gastrointestinal tract can metabolize many components of our daily diet, producing specific metabolites that promote or inhibit tumors. Short-chain fatty acid acetates, cadaverine, butyrate and propionate all inhibit inflammation and tumor development, while secondary bile acids can promote cancerous growths (14, 70). For example, the gut microbiota and its metabolites in mice are affected by dietary cholesterol, which further leads to NAFLD-HCC (15). In addition, diets rich in animal proteins and saturated fats are known to lead to increased bile production, but the entry of bile into the intestine causes specific microflora to produce secondary tumor-promoting bile acids (73). On the contrary, the dietary fiber can be metabolized to short-chain fatty acids by Clostridium perfringens, a branch of colonic bacteria, maintaining normal physiological function of the intestine and maintaining flora balance (74). Butyrate, among the three most abundant short-chain fatty acids, as an energetic substance for colon cells participates in the prevention of colorectal cancer in murine model studies (75).

### Distant sites

Due to altered gut microbiota, the metabolism in the gut may be altered. Immune cells, intestinal microbiota, its metabolites, and cytokines can leave the intestine via the blood circulation and therefore influence tumors occurring in remote areas of the body (76). An extensive MS-based metabolomics study showed a surprisingly large effect of intestinal microbiota on blood metabolites in mammals, specifically on the abundance of some metabolites (77). Some of these specific metabolites promote the development of certain tumors. For instance, intestinal microbiota affects the metabolism of estrogen and thus predisposes to prostate tumors (78). Symbiotic bacteria induce TLR5 and activate NF-κB signaling in B cells in mice carrying K-ras and p53 mutant genes, promoting inflammatory responses and oncogenesis (79). Besides, intestinal bacteria affect brain function and metabolism (36).

### Microbiome-mediated several cancers pathogenesis

**Figure 2:**
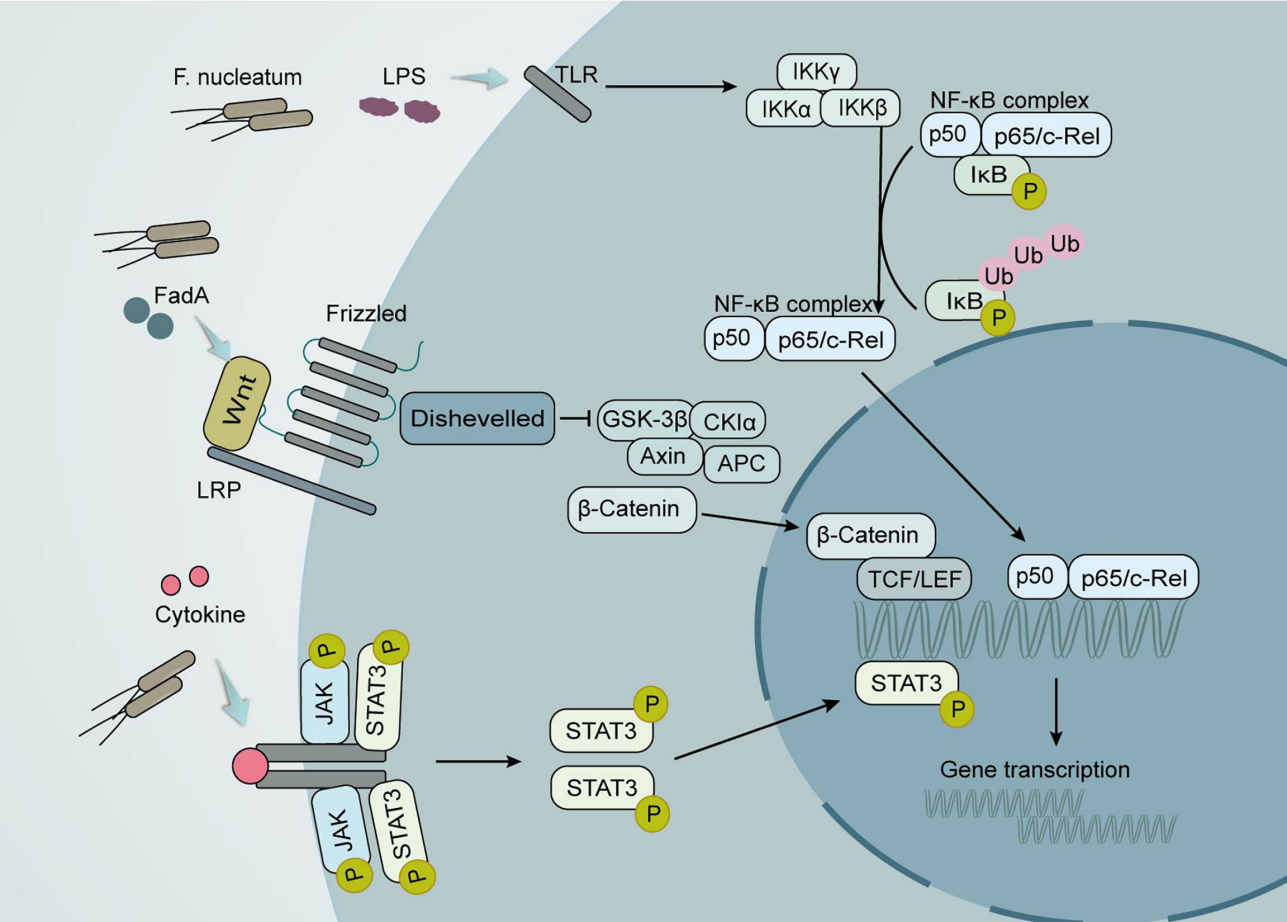
Local effects of the gut microbiota on CRC development. Clostridium nucleatum promotes tumor development through multiple mechanisms. Its production of the virulence factor FadA can lead to increased expression of membrane-linked protein A1 *via* E-calmodulin, which in turn activates Wnt/β-linked protein signaling. The wnt ligand, a secreted glycoprotein, can bind to coiled-coil receptors and form larger cell surface complexes with lipoprotein receptor-associated protein (LRP). Displacement of the multifunctional kinase GSK-3β from the regulatory APC/Axin/GSK-3β complex is triggered by the activated Wnt receptor complex. The stable β-linked protein translocates to the nucleus, displaces the co-inhibitor upon binding to LEF/TCF transcription factors, and recruits the coactivator. Lipopolysaccharide (LPS) in F. nucleatum causes the activation of the nuclear factor κ light chain enhancer (NF-κB) signaling pathway in activated B cells. The conventional signaling pathway is that NF-κB/Rel proteins bind to and are inhibited by IκB proteins. Lipopolysaccharide (LPS) activates IKK complexes (IKKβ, IKKα, and IKKγ) *via* toll-like receptors (TLR) to phosphorylate IκB proteins. IκB phosphorylation leads to its ubiquitination and proteasomal degradation followed by release of NF-κB/Rel complexes. The active NF-κB/Rel complex is further translocated into the nucleus and induces target gene expression. Another important signaling pathway that activates cytokines is the Janus kinase/signal transducer and activator of transcription (JAK-STAT) pathway. When STAT is phosphorylated, it polymerizes into an activated form of transcriptional activator and enters the nucleus to bind to target genes and promote their transcription.

It is worth noting that these mechanisms often co-exist in the course of tumor development (14). More specifically, the disorder of intestinal flora leads to the progression of CRC through different signal pathways (**Figure 2**) (44, 80). Clostridium nucleatum can encode an adhesin, FadA, which then activates β-linked protein signaling and modulates inflammatory and oncogenic responses differentially by binding to lectins and E-calciferin on the surface of host epithelial cells (81). By triggering TLR4 dimerization and recruitment of MyD88 to the receptor, activation of the NF-κB signaling pathway caused by LPS in F.nucleatum is essential for the mediation of the innate vaccine response, consequently producing a proinflammatory microenvironment (82, 83). JAK-STAT is the other signaling pathway that is activated in the development of tumors (62). Apart from different signaling pathways of CRC development, hepatobiliary cancer is also associated with the secretion of AvrA-activated b-linked protein by Salmonella typhi strains (84).

## Modulation of host intestinal microbiota by FMT

**Figure 3:**
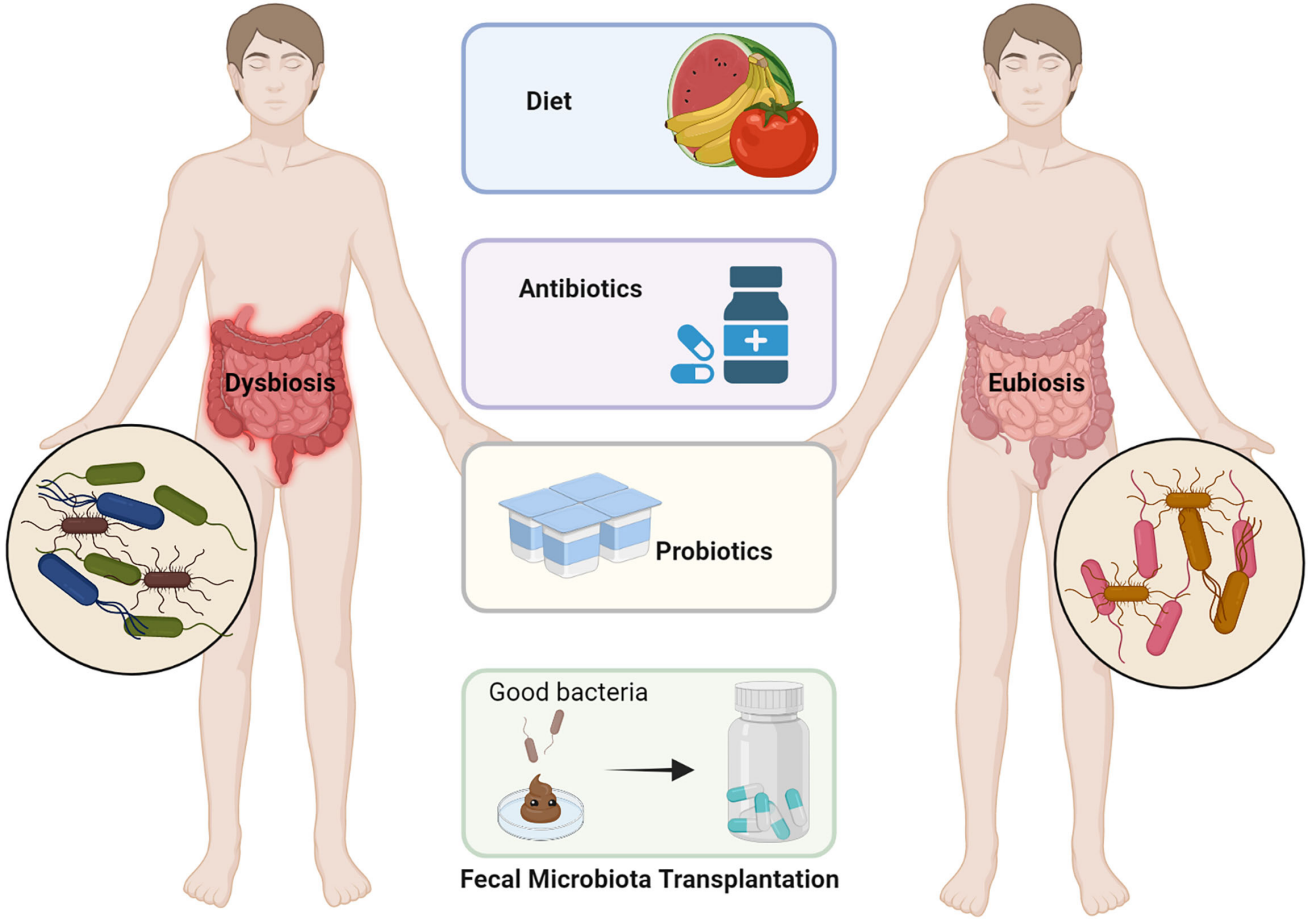
Various approaches for modification of the dysbiotic gut. Dietary modifications such as increasing dietary fiber, antibiotic treatment for pathogenic bacteria, probiotics to increase the colonization of beneficial bacteria in the gut, and fecal microbiome transplantation (FMT) are all options for regulating intestinal flora imbalance (adapted from 16). (The figure was created with Biorender.com).

Experiments in mouse models have confirmed that microbiota can cause cancer and a wide range of other diseases, and microbiota can also be used to therapeutically treat cancer. Dietary interventions, probiotic supplementation, antibiotics, and FMT are a few of the methods currently available to alter the microbiota in the gut and reverse intestinal dysbiosis (**Figure 3**) (16, 85). Consuming dietary fiber-rich foods and prebiotics early in life, minimizing red meat intake, and keeping body weight in the normal range can help reduce the incidence of tumors (86, 87). Similarly, the intake of probiotics can restore intestinal microbial balance and prevent colonic infection (88–90). However, there is a lack of evidence that probiotics can colonize the intestinal mucosa, and especially the role of probiotics in humans after the use of antibiotics is more elusive (91, 92). On the other hand, probiotics also affect the efficacy of ICB therapy in patients with cancer (93). It is no doubt that the administration of antibiotics can suppress or kill pathogenic micro-organisms within the host. Nevertheless, the unregulated use of broad-spectrum antibiotics may lead to antibiotic resistance, which in turn can lead to dysbiosis and even cancer development (94).

**Figure 4:**
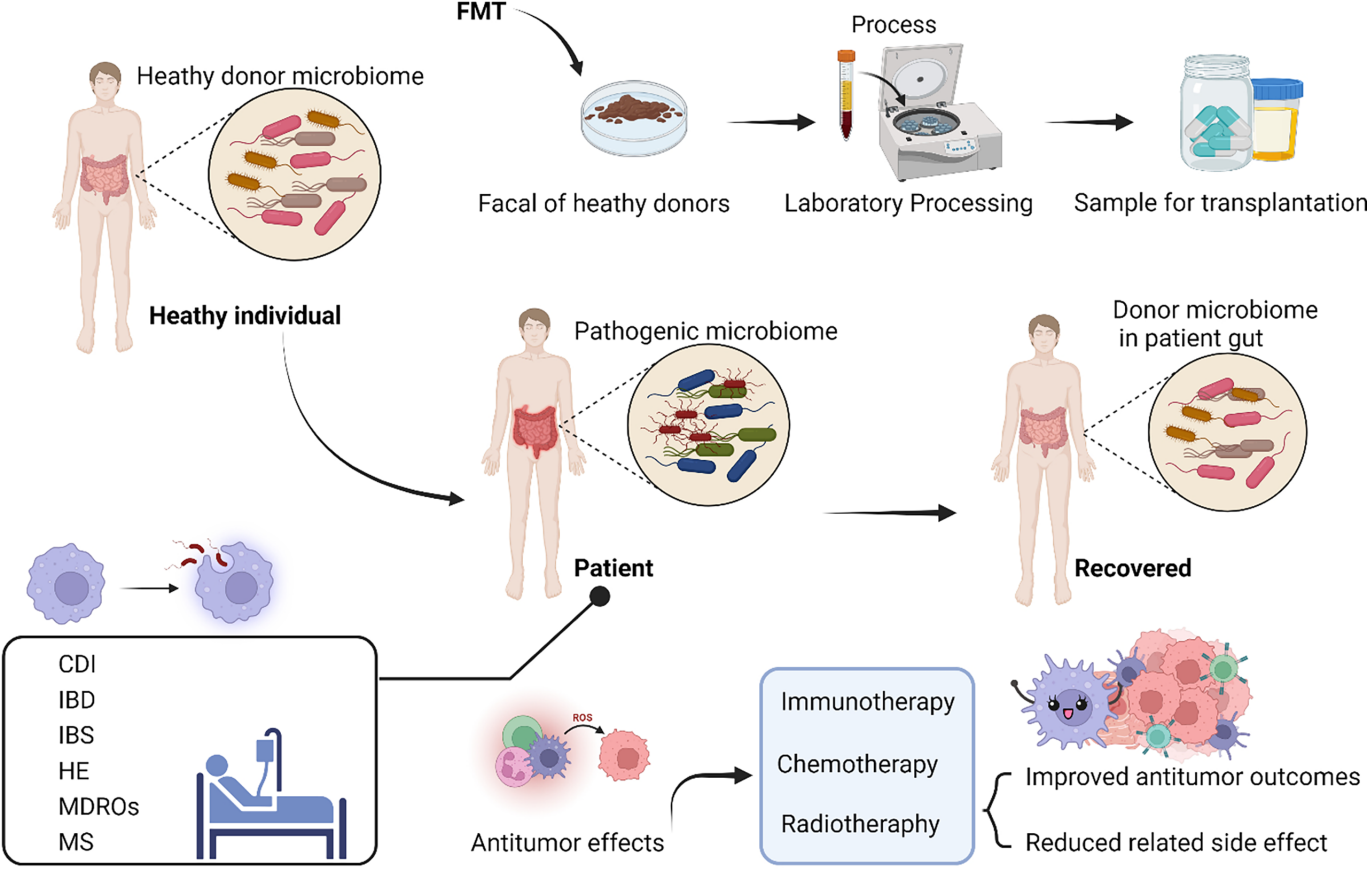
Fecal microbiome transplantation (FMT) from a healthy donor improves the process of dysbiosis and various disorders in patients. FMT can treat many diseases including Clostridioides Difficile infections (CDIs), colonized with multidrug-resistant organisms (MDROs), inflammatory bowel disease (IBD), irritable bowel syndrome (IBS), and neurological disorders including multiple sclerosis (MS), hepatic encephalopathy (HE), Parkinson’s disease, diabetic neuropathy and so on. On the one hand, FMT may improve the gut microbiological environment in patients with insignificant therapeutic efficacy or severe side effects. On the other hand, FMT has antitumor effects and/or reduces the occurrence of associated toxic events (combination FMT with immunotherapy, chemotherapy or radiotherapy). (The figure was created with Biorender.com).

In addition to the above therapies, FMT, which originally originated from TCM theory more than 1700 years ago, has also become an acceptable method of modulating the gut flora to treat disease today (17). In the process of fecal microbiota transplantation, fecal suspensions from healthy individuals are transferred to the patient’s gastrointestinal tract in a variety of ways to re-establish new intestinal flora for the treatment of intestinal and extraintestinal diseases (**Figure 4**) (95). On the one hand, it can be administered through the upper gastrointestinal tract such as the duodenal tube or oral capsules, on the other hand, it can be administered through the lower gastrointestinal tract, by colonoscopy or enema (96–99). Over the last decade, FMT has become relatively mature and received increased attention (100). Until now, FMT has published case reports for many conditions treated, ranging from celiac disease, constipation and cystitis (101–103). More than that, promising results have indicated that FMT also is effective in other digestive diseases, including colonized with MDROs, IBD, IBS, and neurological disorders including MS, HE, Parkinson’s disease, diabetic neuropathy (18, 21–25).

FMT has its unique advantages, as it not only upsurges microbial diversity but also establishes the cross-border balance between intestinal bacteria, viruses and fungi. In a clinical trial, patients with active left UC were randomly divided into FMT or 5-ASA enema groups showed that the gut microbial diversity was at a similar level in both groups, however, FMT remained effective over three months compared to 5-ASA (104). Similarly, a study using single-nucleotide variants in the subgenome monitored the strain population in fecal samples of patients with metabolic syndrome after FMT. It was also found that donor and recipient strains coexisted widely and lasted for 3 months after treatment. Although the success of colonisation of the recipient’s intestine by existing strains was higher than that of the newer species, the latter was also located within the fluctuating levels seen in similar time frames in health individuals (105). These both indicated that the donor bacterial strain persists in the recipient’s intestine for up to 3 months. Therefore, FMT is certainly an efficient means to modulate the host intestinal microbiota, which is considered to be a breakthrough in medical progress in recent years (18, 106). In particular, animal studies in the anti-cancer treatment show that FMT can be widespread applied (107–109). For example, FMT maintains the stability of the intestinal environment and improves DSS-induced colonitis in mice (110). In addition, Rosshart et al. reported that FMT enhanced host resistance to DSS or azomethane-induced colorectal tumorigenesis in 2017 (53).

### Link between the modulatory role of FMT on the host intestinal microbiota and its antitumor effect

There is numerous microbiota in the human body, which regulate physiological functions such as host immunity, metabolism, and cell response. Different gut microbiomes can affect whether the body responds to chemotherapy and immunotherapy when cancer is present (35, 51, 111, 112). Growing evidence that the intestinal microbiota can improve the efficiency or reduce the toxicities of antineoplastic treatments (chemotherapy, immunotherapy and radiotherapy) (113, 114). In addition, FMT has made breakthroughs in the anti-tumor field by modulating the host gut microbiota as a promising approach to the management and prevention of multiple cancers (108, 115, 116).

### Emerging use of FMT in tumour immunotherapy

Gut microbes can regulate host efficacy to anti-cancer medicines and immune regulation is one of the core factors to promote these differential responses (117, 118). Immune checkpoint blockades (ICBs), including antibodies to PD‐1, PD-L1, and CTLA-4, have been licensed by FDA and have been clinically effective against most types of cancer, such as malignant melanoma (119). The variability in therapeutic responses among patients and even the failure of response in certain types of tumors remain of great concern (120). In addition,ICBs can induce immune-related adverse reactions, especially the response of colitis and pituitary gland inflammation to CTLA-4 antibody, as well as thyroid dysfunction and pneumonia after blocking PD-1-PD-L1 interaction. There are many factors related to the efficacy and toxicity of ICB cancer treatment (121), and the different resistance responses of people to ICIs are in part linked to the different components of the gut flora. Different gut microbiota composition of patients has been shown to modulate host efficacy to anti-CTLA-4 or anti-PD-1/PD-L1 immunotherapy (35, 45, 111, 122c; 123). Antibodies against CTLA-4 have been used successfully in clinical practice for immunotherapy of tumors. Studies have shown that the anti-tumor action of CTLA-4 blockers depends on different bacteriophage types (124–126), and that CTLA-4 antibody treatment of melanoma patients favors the growth of Bifidobacterium fragilis, which has anti-cancer properties (112). Moreover, studies found that in sterile or antibiotic-treated mice, FMT in patients with cancer responding to ICIs may improve the antitumor action of PD-1 blockers, while FMT in patients who did not respond cannot improve the antitumor action of PD-1 blockers (108). In the majority of cases, FMT can transform the non-responder of cancer immunotherapy into a responder, that is, FMT can affect the efficacy of ICB and be used in immunotherapy (127). Encouragingly, this has been demonstrated in three recent reports in which sterile mice and mice treated with antibiotics showed an increased response to anti-PD-1 treatment after receiving a rigorously screened fecal transplantation of FMT (108, 128, 129). Another study showed that patients who were treated with broad spectrum antibiotics were less effective with anti-PD-1 (108). Besides, there is a complex relationship between intestinal microbiota and cancer immunotherapy response (130). The gut microbiota has been associated with ICI response, however cohort dependent (131). Based on these observations, clinical interventions to restore favorable microbiota are underway.

Recent research has proved that FMT combined with anti-PD-1 therapy is potentially efficacious in treating refractory metastatic melanoma. FMT is being evaluated for its potential to enhance immune checkpoint blockade therapy by some clinical studies (mainly for patients with metastatic melanoma) (**Table 1**). Trials for prostate cancer, gastrointestinal cancer, lung cancer, and mesothelioma are also ongoing. Most clinical trials use patients with good results for anti-PD-1 as stool donors, from which eligible stool samples are selected. For example, an ongoing clinical trial is evaluating the safety and feasibility of FMT in combination with anti-PD-1 immunotherapy (NCT03353402). Besides, a University of Pittsburgh Phase 2 clinical trial is evaluating the effect of FMT combined with pembrolizumab in patients suffering from melanoma who are not responding to anti-PD-1 therapy (NCT03341143). Additional clinical trials are also underway to examine the effects of incremental FMT in people with melanoma or genitourinary cancers (NCT03772899, NCT03819296, NCT04577729, NCT04116775, NCT04758507). These clinical studies are of great significance for the modulatory effect of FMT in cancer treatment.

**Table 1:** Ongoing cancer clinical trials investigating FMT and immunotherapy.

Cancer Type	Intervention	Investigator	Status	Identifer	Phase
Melanoma	FMT + ICIs	Lawson Health Research Institute	Active, not recruiting	NCT03772899	Phase 1
Advanced Melanoma	FMT (via colonoscopy and stool capsules) + ICIs	Sheba Medical Center	Unknown	NCT03353402	Phase 1
Melanoma	FMT (via colonoscopy) + ICIs	UPMC Hillman Cancer Center	Active, not recruiting	NCT03341143	Phase 2
Melanoma	Autologous or Allogeneic FMT + ICIs	Medical University of Graz	Recruiting	NCT04577729	Not Applicable
Metastatic NSCLC Advanced Melanoma	FMT + ICIs	Centre hospitalier de l’Université de Montréal	Recruiting	NCT04951583	Phase 2
Advanced Melanoma	FMT + ICIs	The Netherlands Cancer Institute	Not yet recruiting	NCT05251389	Phase 1/2
Melanoma	FMT + ICIs	Assistance Publique Hôpitaux de Paris	Recruiting	NCT04988841	Phase 2
Advanced Melanoma	FMT + ICIs	Oslo University Hospital	Recruiting	NCT05286294	Phase 2
Melanoma	FMT + ICIs	M.D. Anderson Cancer Center	Recruiting	NCT03819296	Phase 1/2
Melanoma NSCLC	FMT (via stool capsules) + ICIs	Sheba Medical Center	Recruiting	NCT04521075	Phase 1/2
Gastrointestinal Cancer	FMT (via stool capsules) + ICIs	Peking University	Recruiting	NCT04130763	Phase 1
Malignant Colorectal Neoplasms	FMT + ICIs + Sintilimab and Fruquintinib	Chinese Academy of Medical Sciences	Not yet recruiting	NCT05279677	Phase 2
Metastatic Colorectal Adenocarcinoma	FMT (via stool capsules) + ICIs	M.D. Anderson Cancer Center	Recruiting	NCT04729322	Early Phase 1
Mesothelioma	FMT (via colonoscopy) + ICIs	ProgenaBiome	Completed	NCT04056026	Early Phase 1
Solid Malignancies	FMT (via colonoscopy) + ICIs	Michael Scharl, University of Zurich	Recruiting	NCT05273255	Not Applicable
Solid Carcinoma	FMT + ICIs	Asan Medical Center	Recruiting	NCT04264975	Not Applicable
Malignant Genitourinary System Neoplasm	FMT (via colonoscopy) + ICIs	M.D. Anderson Cancer Center	Recruiting	NCT04038619	Phase 1
NSCLC	FMT (via stool capsules) + ICIs	Shanghai Zhongshan Hospital	Not yet recruiting	NCT05008861	Phase 1
Advanced Lung Cancer	FMT (via stool capsules) + ICIs	Fundacion para la Investigacion Biomedica del Hospital Universitario Ramon y Cajal	Recruiting	NCT04924374	Not Applicable
Renal Cell Carcinoma	FMT (via stool capsules) + ICIs	Fondazione Policlinico Universitario Agostino Gemelli IRCCS	Recruiting	NCT04758507	Phase 1/2
Prostate Cancer	FMT(via endoscopy)+ ICIs + Enzalutamide	VA Portland Health Care System	Recruiting	NCT04116775	Phase 2

## Other clinical applications of FMT in antitumor effect

Chemotherapy is a common method of treating cancer (132). Microorganisms and chemotherapeutic agents such as 5-fluorouracil and cyclophosphamide can interact with each other in both directions. On one hand, chemotherapy alters the composition of the patient’s microbial community, which may cause serious side effects in immunocompromised cancer patients (133, 134). On the other hand, the microbiome can metabolize drugs and modify anticancer drug efficacy (16, 135a; 136b). There is growing evidence that improving drug efficacy, increasing antitumor effects, and reducing toxic effects are the three main effects of intestinal microbes on chemotherapeutic drugs (111). Cyclophosphamide is an alkylating agent used in cancer therapy and is widely used in the treatment of leukemia, lymphoma, multiple myeloma, rheumatoid arthritis, and before bone marrow transplantation (109, 134). Gram-negative bacilli increase the antitumor effect of cyclophosphamide by increasing T cells infiltration in tumor sites (115, 137). In certain settings, the microbiota may also become a drug target to improve the side effects of many chemotherapeutic drugs on the gastrointestinal tract. Although the underlying molecular and cellular mechanisms of FMT are unknown, it may involve direct donor-intestinal microbiota-host interactions, thereby mediating the observed effects on host physiology, the intestinal mucosal barrier, and the immune system (138). In addition, animal experiments have shown that FMT improves the recovery rate of chemotherapy mice (139). Thus, FMT can effectively control the intestinal microbiota, enhance the efficacy of chemotherapy drugs, and reduce inflammation and toxic reactions.

Radiation therapy also performs an irreplaceable part in the treatment of many types of cancer (140). However, exposure to radiation can also damage healthy surrounding tissues (141, 142). Intestinal microbes have a key role in radiation-induced intestinal damage (143). In a mouse model, gavage of intestinal microorganisms attenuated and protected against radiation-induced injury. Specifically, FMT was able to improve irradiated mice recovery rate (107). The results of the current study suggest that gut microbial transplantation-mediated fluctuations in lncRNA expression profiles may play an important role in mitigating radiation-induced injury, which warrants further investigation. Thus, as a potential treatment to mitigate radiation toxicity, FMT may be used in oncologic radiotherapy to improve prognosis (107).

## Modern perspectives on the application of FMT

Although FMT is safe and easy to implement, concerns have been raised after a patient died from the procedure. The cause of death in patients receiving FMT is an invasive multi-drug resistant E. coli infection, although this E. coli is inherent in the feces of healthy donors (144). Identifying such pathogenic species and understanding mechanisms that promote their cohabitation is necessary for the development of effective and safe microbiome-based therapies, as exemplified in the present study (145). The composition of intestinal flora varies from patient to patient, and the effect of FMT treatment varies (146), which also suggests that the long-term impact of FMT not yet known and should be used with caution. For example, studies have shown that rumenococci affect the efficacy of FMT since donors with higher levels of rumenococci are more likely to fail the procedure (110). Meanwhile, when FMT is considered a treatment option, its safety remains an important issue (147). In a clinical trial on the safety of FMT, adverse effects such as abdominal pain, cramping or pressure, diarrhea, or constipation were reported in about 20% of patients in the FMT group, in contrast to an average of 2% in those on placebo. Moreover, two patients developed diverticulitis compared with none on placebo (148). It will be essential to conduct future controlled research studies exploring the safe, duration, dosing, formulation, administration route, and combinations of FMT to determine whether FMT can be used for cancer treatment (149).

When relevant institutions do stool testing of donors, drug-resistant bacteria screening must be performed (150). Recipients most at risk for adverse events are patients with poor immune status and severe intestinal ulcers. Patients with severe immune deficiencies should be first excluded. The U.S. FDA has mandated updated screening guidelines for fecal donations for FMT and requires public fecal banks to update their screening to ensure that all FMTs are properly screened for these pathogens (151). Eligible healthy donors are screened primarily through the following eight areas: age, physiology, pathology, psychology, accuracy, timing, life circumstances, and recipient (152, 153). The safety of FMT also requires long-term follow-up. With the current increasing emphasis on FMT therapy, standardized procedures in laboratories and in clinical working processes are essential to ensure the importance of the graft microbiota, the efficiency of FMT, and the reduction of underlying risks (116). To ensure the safe delivery of FMT and to provide appropriate access for those who require it, we recommend the use of fecal banks, such as NDFB, in order to meet the basic criteria to accomplish simple, secure, and cost effective FMT treatment (154). At the same time, FMT protocols should be optimized and standardized for different indications to further determine the long-term security of FMT. More importantly, COVID-19 caused by SARS-CoV-2 infection is still prevalent worldwide currently (155). It has recently been reported that SARS-Cov-2 RNA has also been detectable in the feces of infected individuals (156, 157), which suggests that it is essential to screen for COVID-19 before performing FMT.

Shortly, the microbiota will be an important asset in the diagnosis and FMT will also be widely used as a treatment for human diseases (146, 158). Autologous fecal microbiota transplantation (a-FMT) is also a form of FMT, which means the storage of human feces in a healthy state as an alternative to avoid pathogenic allogeneic feces or related risks and is used to restore the normal function of the original intestinal flora after the dysbiosis of its intestinal flora. Compared to common FMT, which has been extensively studied, a-FMT may be an underestimated effective treatment and exhibit long-term efficacy (159). Besides, with the rise of the selective microbiota transplantation (SMT) concept, further research should focus on identifying the precise strains and specific functions, and the SMT effect of two or more strains may be the emerging direction (116, 160). Colonic TET is a new form of FMT or SMT administration that not only facilitates patient access but also improves patient outcomes. Choosing the appropriate FMT delivery method according to the patient’s specific situation can meet the patient’s needs and reduce side effects. Towards this objective, the state-of-the-art and logical delivery method for MT is the colonic transendoscopic tube (TET). Colonic TET as a new modality for FMT or SMT administration that not only facilitates patient access but also improves patient outcomes (161, 162).

## Conclusions

Using the gut microbiota as an adjuvant to anti-cancer treatment has attracted the interest of researchers in recent years. Patient response to anticancer therapy and the incidence of adverse events is related to the gut microbiota. Accumulating evidence emphasizes that FMT can improve the efficacy of chemotherapeutic agents and reduce related undesirable events. In oncology patients, toxicity associated with radiotherapy is often accompanied by a structured microbial community. Similarly, the type and amount of gut microbiota have a non-negligible effect on the host’s reaction to anti-PD-1 and anti-CTLA-4 immunotherapies. The combination of FMT with anti-PD-1 for the treatment of refractory metastatic melanomahas recently been shown to be safe, practical, and potentially effective. Several clinical studies in patients with advanced melanoma are assessing the potential of FMT-enhanced immune checkpoint blockade therapy. Overall, FMT is a hopeful method to improve the therapeutic effect of immunotherapy while reducing the side effects of chemotherapy by modulating the gut microbiota. In future studies, it is necessary to explore the safe, duration, dosing, formulation, administration route, and combinations of FMT to determine the optimal regimen of it for cancer treatment.

## Author contributions

HX, ZL, and YR provided direction and guidance throughout the preparation of this manuscript. CC, HX and YR wrote and edited the manuscript. JR reviewed and made significant revisions to the manuscript. HX, SW, LL, CG, LW and ZL collected and prepared the related papers. All authors contributed to the article and approved the submitted version.

## Conflict of interest

The authors declare that the research was conducted in the absence of any commercial or financial relationships that could be construed as a potential conflict of interest.

## Publisher’s note

All claims expressed in this article are solely those of the authors and do not necessarily represent those of their affiliated organizations, or those of the publisher, the editors and the reviewers. Any product that may be evaluated in this article, or claim that may be made by its manufacturer, is not guaranteed or endorsed by the publisher.

## Abbreviations

FMT, Fecal microbiota transplantation; HE, Hepatic encephalopathy; CDIs, Clostridioides Difficile infections; CRC, Colorectal cancer; HPV, Human papilloma virus; NSCLC, Non-Small Cell Lung Cancer; HBV,Hepatitis B virus; SFB, Segmented filamentous bacteria; HCV, Hepatitis C virus; ICBs, Immune checkpoint blockades; MDROs, Multidrug-resistant organisms; a-FMT, Autologous fecal microbiota transplantation; IBD, Inflammatory bowel disease; SMT, Selective microbiota transplantation; IBS, Irritable bowel syndrome; TET, Transendoscopic enteral tubing; MS Multiple sclerosis.
